# *Plasmodium falciparum* Antigen 332 Is a Resident Peripheral Membrane Protein of Maurer's Clefts

**DOI:** 10.1371/journal.pone.0046980

**Published:** 2012-11-20

**Authors:** Sandra Nilsson, Davide Angeletti, Mats Wahlgren, Qijun Chen, Kirsten Moll

**Affiliations:** 1 Department of Microbiology, Tumor and Cell Biology, Karolinska Institutet, Stockholm, Sweden; 2 Laboratory of Parasitology, Institute of Pathogen Biology, Chinese Academy of Medical Sciences, Beijing, People's Republic of China; Bernhard Nocht Institute for Tropical Medicine, Germany

## Abstract

During the intraerythrocytic development of *Plasmodium falciparum*, the malaria parasite remodels the host cell cytosol by inducing membranous structures termed Maurer's clefts and inserting parasite proteins into the red blood cell cytoskeleton and plasma membrane. Pf332 is the largest known asexual malaria antigen that is exported into the red blood cell cytosol where it associates with Maurer's clefts. In the current work, we have utilized a set of different biochemical assays to analyze the solubility of the endogenous Pf332 molecule during its trafficking from the endoplasmic reticulum within the parasite to the host cell cytosol. Solubilization studies demonstrate that Pf332 is synthesized and trafficked within the parasite as a peripheral membrane protein, which after export into the host cell cytosol associates with the cytoplasmic side of Maurer's clefts in a peripheral manner. By immunofluorescence microscopy and flow cytometry, we show that Pf332 persists in close association with Maurer's clefts throughout trophozoite maturation and schizogony, and does not become exposed at the host cell surface. Our data also indicate that Pf332 interacts with the host cell cytoskeleton, but only in very mature parasite stages. Thus, the present study describes Pf332 as a resident peripheral membrane protein of Maurer's clefts and suggests that the antigen participates in host cytoskeleton modifications at completion of the intraerythrocytic developmental cycle.

## Introduction

*Plasmodium falciparum* parasites cause the most severe form of malaria with over 225 million clinical cases leading to approximately 800 000 deaths every year [1]. More than 80% of these deaths take place in sub-Saharan Africa and most are among children under five years of age [1].

Following invasion of human red blood cells (RBC), the malaria parasite resides within a membrane-enclosed parasitophorous vacuole (PV). In contrast to unparasitized RBC, parasitized RBC (pRBC) are poorly deformable, highly rigid, and display the propensity to adhere to vascular endothelial cells and unparasitized RBC [2], [3]. The increased rigidity and adhesiveness of pRBC result in their accumulation in the microvasculature of various organs and play an important role in malaria pathogenesis. At a molecular level, these host cell modifications are mediated by a subset of parasite-derived proteins that are exported across the PV membrane (PVM) into the RBC cytosol where they interact with the host cell cytoskeleton or are exposed at the RBC surface [4], [5]. Cytoskeleton binding proteins such as knob-associated histidine-rich protein (KAHRP) [6], [7], *P. falciparum* erythrocyte membrane protein 3 (PfEMP3) [8], mature parasite-infected erythrocyte surface antigen (MESA) [9], [10], and ring parasite-infected erythrocyte surface antigen (RESA) [11], [12] are known to be responsible for the increased rigidity of pRBC, whereas the virulence and surface associated *P. falciparum* erythrocyte membrane protein 1 (PfEMP1) is a known mediator of the adhesive phenotype [13], [14], [15].

Since the RBC is a transcriptionally and translationally inactive cell that lacks a protein secretory apparatus generally present in other eukaryotic cells, the parasite must establish its own trafficking-machinery responsible for protein export [4]. These parasite-induced modifications include the formation of flattened membranous structures termed Maurer's clefts [16], [17], which are believed to be important intermediate compartments involved in sorting and trafficking of parasite proteins destined to the RBC plasma membrane [18]. Several exported parasite proteins that are resident to Maurer's clefts have been described, including skeleton binding protein 1 (SBP1) [19], membrane-associated histidine-rich protein 1 (MAHRP1) [20], and ring exported protein 1 and 2 (REX1 and REX2) [21], [22]. Although many exported proteins contain a signal sequence that directs the protein into the secretory pathway and a short amino acid motif termed the *Plasmodium* export element (PEXEL) or vacuolar transport signal (VTS) that direct the antigen onwards [23], [24], these Maurer's clefts residents lack both [21], [25], [26], [27].

**Figure 1:**
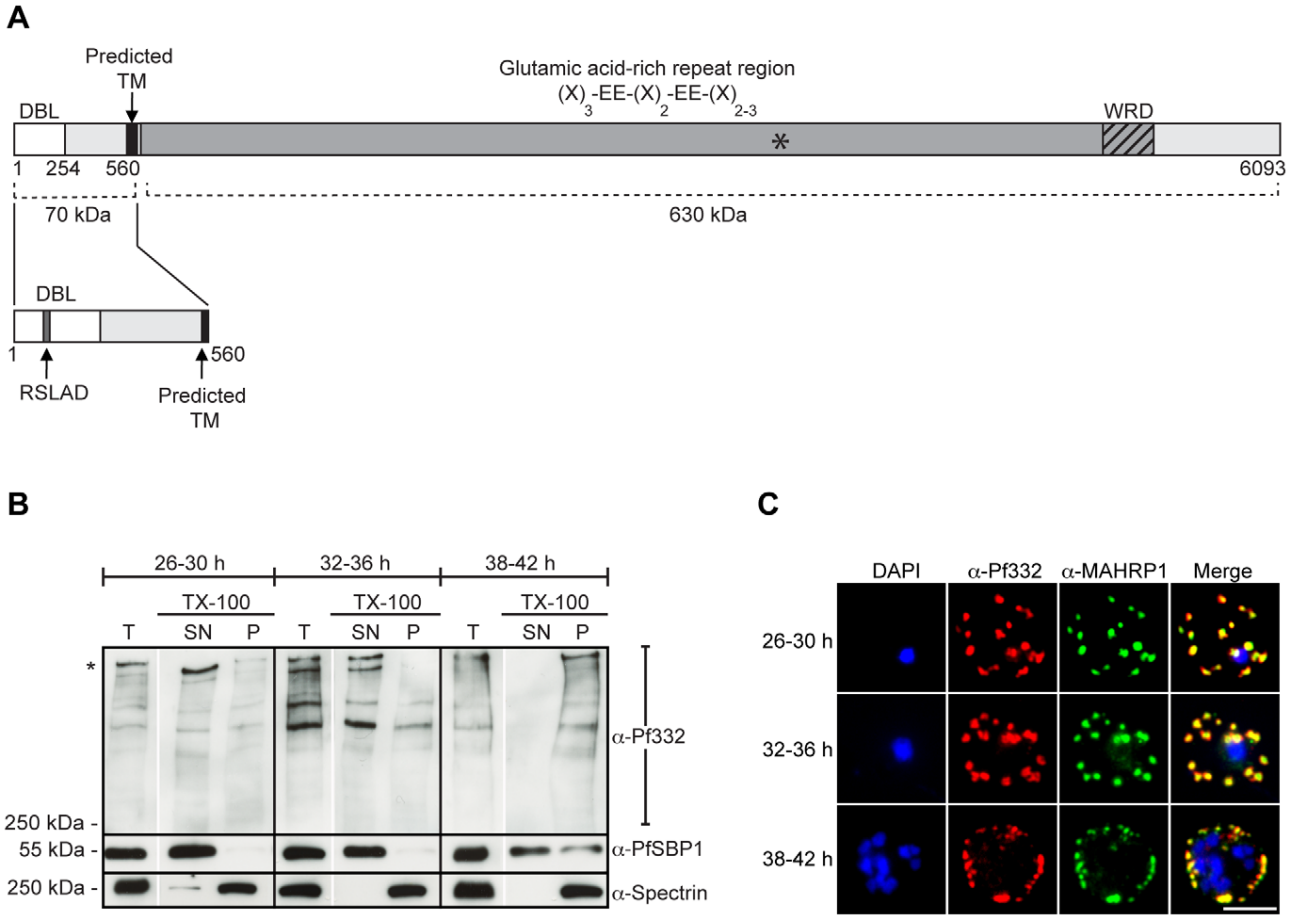
Pf332 becomes increasingly insoluble in Triton X-100 as the parasite matures. (**A**) Schematic representation of full-length Pf332. Residues 1–570 are encoded by the first exon, which contains a DBL domain (white), a PEXEL-like motif (RSLAD), and a predicted TM (black). The second exon encodes an extensive glutamic acid-rich repeat region with the consensus (X)_3_-EE-(X)_2_-EE-(X)_2–3_ (dark grey; E: glutamic acid, X: hydrophobic amino acid) followed by a tryptophan-rich domain (WRD; striped). The location of the recognition site for the Pf332-EB200 antibody is marked with an asterisk. The complete Pf332 protein comprises approximately 700 kDa, of which the DBL domain and the predicted TM region constitute 70 kDa. (**B**) Western blot analysis of pRBC extracted with TX-100 at different time-points during parasite development. Extracted material was separated by centrifugation into a supernatant fraction (SN, containing proteins soluble in water or TX-100) and a pellet fraction (P, containing TX-100 insoluble proteins). Blots were probed with monoclonal anti-Pf332-DBL, polyclonal anti-PfSBP1, and monoclonal anti-spectrin antibodies. The first lane at each time-point (T) represents total non-extracted pRBC lysate. Full-length Pf332 is marked with an asterisk. The experiment was repeated on three separate occasions, and blots shown are from the same individual experiment. Each lane represents protein extract from 3.3×10^6^ pRBC. (**C**) IFA of air-dried monolayers of pRBC collected at each of the three aforementioned time-points. Slides were probed with mouse monoclonal anti-Pf332-DBL (red) and rabbit polyclonal anti-PfMAHRP1 antibodies (green). The parasite was counterstained with DAPI (blue). Scale bar indicates 5 µm. (DBL: Duffy binding-like, TM: transmembrane region, TX-100: Triton X-100, IFA: immunofluorescence microscopy assay).

The 700 kDa Pf332 molecule is the largest known exported asexual malaria protein [28]. The antigen consists of an N-terminal Duffy binding-like (DBL) domain followed by a putative transmembrane region, and a large number of negatively charged repeats that are not identical but have the consensus (X)_3_-EE-(X)_2_-EE-(X)_2–3_, where E is glutamic acid (Glu) and X is a hydrophobic amino acid (Figure 1A) [29], [30]. Together, the Glu-rich repeats make up more than 90% of the protein. Although Pf332 is exported into the host cell cytosol, it lacks both a canonical signal sequence and a classical PEXEL motif. Pf332 does, however, contain a PEXEL-like sequence (RSLAD) starting 78 amino acids from the N-terminus [30]. Within the host cell cytosol, Pf332 can be found in close association with Maurer's clefts [31], [32]; however, it is not clear whether this is a permanent or a transient localization of the antigen, as Pf332 has been described to also associate with the RBC plasma membrane [29], [30], [32]. The function of Pf332 is not well characterized, but host cells parasitized with a *Pf332* knockout strain display an increased rigidity compared to RBC parasitized with wild type parasites, indicating that the antigen interacts with the RBC cytoskeleton [33].

Since a biochemical characterization of Pf332 and its membrane associations is currently lacking, we have investigated the solubility characteristics of the endogenous Pf332 molecule during the intraerythrocytic development of *P. falciparum*. We here report that Pf332 is synthesized as a peripheral membrane protein, which after export into the host cell cytosol associates with the cytoplasmic side of Maurer's clefts in a peripheral manner throughout schizogony. Pf332 does not become exposed at the RBC surface, but becomes increasingly detergent insoluble as the parasite matures, a phenomenon that most likely results from interactions between Pf332 and the host cell cytoskeleton. Gaining more insight into host cell modifications exerted by *P. falciparum* is essential for increasing our understanding of malaria pathogenesis, and may lead to novel intervention strategies for controlling malaria.

## Results

### Pf332 displays varying solubility characteristics in the host cell cytosol

The Pf332 protein can first be detected within the parasite at 20–24 h post invasion (p.i.), after which it translocates across the PVM into the host cell cytosol [30], [31]. In order to study the solubility characteristics of Pf332 within the RBC cytosol, we conducted a time-course study where enriched HB3 pRBC were harvested at 26–30, 32–36, and 38–42 h p.i and lysed with Triton X-100 (TX-100). The resulting protein extracts were separated by centrifugation into a TX-100 soluble fraction containing water-soluble and membrane associated proteins (supernatant/SN), and a TX-100 insoluble/sodium dodecyl sulfate (SDS) soluble fraction containing cytoskeleton proteins (pellet/P). Extracts were analyzed by Western blot, and membranes probed with antibodies towards Pf332-DBL and two markers for different solubility profiles: SBP1 (an integral membrane protein of Maurer's clefts that is soluble in TX-100) and spectrin (a peripheral cytoskeleton protein that is insoluble in TX-100). SBP1 is also known to interact with the cytoskeleton in very mature parasite stages (>40 h p.i.) at which time the protein becomes TX-100 insoluble [25], [34].

At 26–30 and 32–36 h p.i., Pf332 was observed almost exclusively in the detergent soluble supernatant and only a small amount of protein was visible in the detergent insoluble pellet (Figure 1B). In contrast, at schizont-stage (38–42 h p.i.), Pf332 was recovered entirely in the insoluble pellet, indicating that the antigen was interacting with the host cell cytoskeleton at completion of the intraerythrocytic developmental cycle. SBP1 displayed a typical profile of an integral membrane protein as it was exclusively recovered in the detergent soluble fraction, although we found some SBP1 also in the detergent insoluble fraction at schizont-stage. As expected, spectrin was retained in the insoluble cytoskeleton fraction at all time-points (Figure 1B).

Several smaller sized Pf332 bands could be observed at all three time-points (Figure 1B) and this pattern has previously been described for Pf332 [33], [35]. The exact mechanism behind their appearance is unclear, but they are likely products of full-length protein processing. At trophozoite-stage, the Pf332 fragments were mainly found in the detergent soluble fraction, whereas they were present in the insoluble fraction at schizont-stage, suggesting that also the processed Pf332 products were interacting with the cytoskeleton in very mature parasite stages. Since the Pf332 fragments were of high molecular weight and could be identified using an antibody towards the N-terminus, we can conclude that they contained the DBL-domain, the predicted transmembrane domain, and the major part of the Glu-rich repeats.

To be able to correlate the observed biochemical profile with the subcellular localization of Pf332, we collected pRBC for immunofluorescence microscopy assays (IFA) at each time-point. Monolayers of pRBC were probed with antibodies towards Pf332-DBL and a well-known Maurer's clefts marker; MAHRP1 [20]. At all three time-points, Pf332 was visualized in Maurer's clefts as demonstrated by the co-localization with MAHRP1, which argues for Pf332 to be a membrane associated protein of Maurer's clefts throughout the mature stages of parasite development (Figure 1C). At 26–30 and 32–36 h p.i., Maurer's clefts were visualized throughout the host cell cytosol, but when the parasite had reached schizont-stage (38–42 h p.i.) and Pf332 was retained in the insoluble cytoskeleton fraction as determined by Western blot (Figure 1B), Maurer's clefts were observed in close proximity to the RBC plasma membrane (Figure 1C).

**Figure 2:**
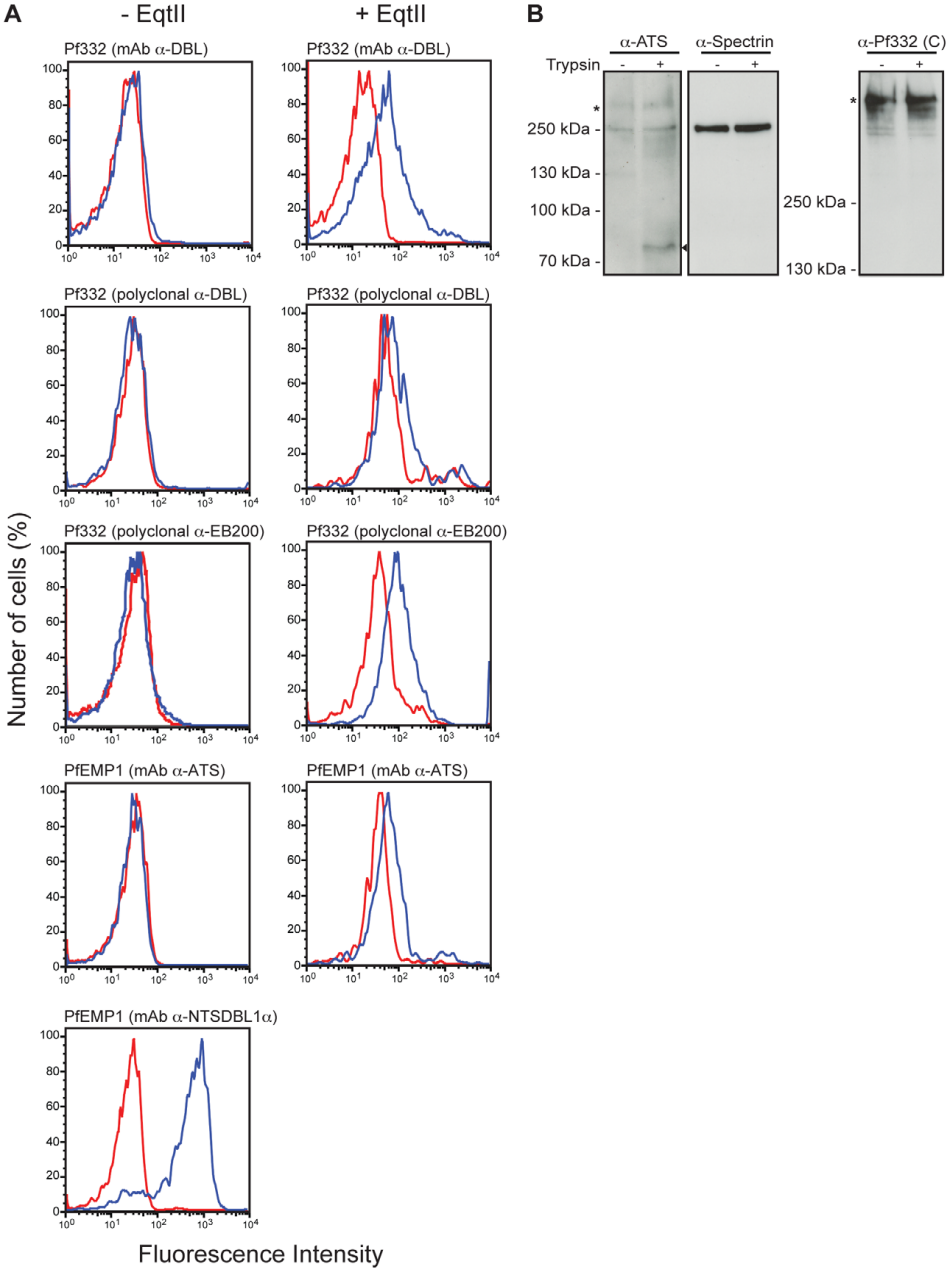
Pf332 is not exposed at the red blood cell surface. (**A**) Surface expression of Pf332 was studied in live intact (left panel) or RBC plasma membrane permeabilized (EqtII-treated, right panel) schizont-stage FCR3S1.2 pRBC by flow cytometry. To detect Pf332, monoclonal mouse anti-Pf332-DBL, polyclonal rat anti-Pf332-DBL (N-terminus of Pf332), and polyclonal rabbit anti-Pf332-EB200 (C-terminus of Pf332) were used. As a marker for a positive surface expression, a monoclonal mouse antibody towards the ectodomain of the PfEMP1 variant expressed by the FCR3S1.2 parasite strain was used (anti-PfEMP1-NTSDBL1α). As a marker for an intracellular localization, a mouse monoclonal antibody towards the intracellular acidic terminal segment (ATS) of PfEMP1 was used. Non-immune mouse immunoglobulin G (IgG) and pre-immune rabbit/rat sera were used as negative controls and are displayed in red. Specific anti-Pf332 and anti-PfEMP1 antibodies are displayed in blue. (**B**) Intact schizont-stage HB3 pRBC were treated with (+) or without (−) trypsin, and whole cell lysates were analyzed by Western blotting using monoclonal anti-PfEMP1-ATS (left) or polyclonal anti-Pf332 antibodies (C-terminus, right). Full-length PfEMP1 and Pf332 are indicated with asterisks. Surface-exposed PfEMP1 is cleaved by trypsin, resulting in a truncated polypeptide migrating at approximately 80 kDa (black arrow head). The blot was also probed with monoclonal mouse anti-spectrin antibodies to confirm equal loading and to show that the anti-PfEMP1-ATS antibody cross-reacts with spectrin (middle).

PfEMP1, the most well-characterized exported parasite protein to date, has been described to be trafficked as a TX-100 soluble protein that only after being incorporated into knob structures and exposed at the RBC surface becomes TX-100 insoluble/SDS-soluble [36], [37]. In order to investigate whether the detergent insolubility of Pf332 at schizont-stage was a result of a surface exposed population of the antigen, we performed flow cytometric analysis on live intact schizont-stage FCR3S1.2 pRBC at 38–42 h p.i. To detect Pf332, we used antibodies directed against the N-terminus (monoclonal anti-Pf332-DBL and polyclonal anti-Pf332-DBL), and the C-terminus (polyclonal anti-Pf332-EB200). For comparison, we used a set of PfEMP1 antibodies against the N-terminal ectodomain of the PfEMP1 variant expressed by the FCR3S1.2 parasite strain (PfEMP1-NTSDBL1α), and the conserved and intracellular C-terminus (acidic terminal segment; PfEMP1-ATS). The majority of pRBC stained positively for PfEMP1 when using the PfEMP1-NTSDBL1α antibody, whereas none of the cells stained positively when using antibodies towards Pf332 or PfEMP1-ATS (Figure 2A, left panel). In contrast, after selectively permeabilizing the RBC plasma membrane with the pore-forming cytolysin Equinatoxin II (EqtII) [38], [39], a large proportion of pRBC stained positively for both Pf332 and PfEMP1-ATS (Figure 2A, right panel). It should be noted that we were unable to detect any surface exposed Pf332 molecules when performing a flow cytometry analysis of live HB3 pRBC using anti-Pf332-DBL antibodies at early trophozoite, late trophozoite, early schizont, and late schizont-stage (Figure S1).

To further confirm the observed intracellular localization of Pf332, we employed a well-established digestion assay that allows for detection of surface exposed molecules due to their sensitivities to trypsin cleavage [36], [40]. Intact schizont-stage HB3 pRBC were treated with (+) or without trypsin (−), and whole cell lysates were analyzed by Western blot. In untreated cells, the PfEMP1-ATS antibody detected only full-length protein migrating at approximately 280 kDa (representing surface exposed and intracellular PfEMP1; Figure 2B). In contrast, in trypsin-treated cells, the PfEMP1-ATS antibody detected both full-length PfEMP1 (representing the intracellular pool) and a truncated product migrating at approximately 80 kDa (representing the protected transmembrane region and ATS-domain of surface exposed PfEMP1; Figure 2B).

If the DBL-domain of Pf332 was exposed at the RBC surface, trypsin treatment would result in a truncated product migrating at approximately 630 kDa, whereas this band would be missing in untreated cells. However, although smaller sized Pf332 fragments were visible in trypsin-treated cells, untreated cells displayed a similar pattern (Figure 2B). Consequently, Pf332 does not appear to be exposed at the RBC surface.

### Pf332 can be extracted by urea and sodium carbonate in trophozoite and schizont-stage pRBC

Although Pf332 displayed a close association with Maurer's clefts throughout trophozoite maturation and schizogony (Figure 1C), it was not clear to us whether the protein was integrated into the lipid bilayer or peripherally attached to the cytoplasmic face of the clefts. In order to obtain information of the mode of attachment of Pf332 to Maurer's clefts, we performed differential extractions of pRBC at both trophozoite (26–30 h p.i.) and schizont-stage (38–42 h p.i.). Water-soluble and membrane fractions were prepared by hypotonic lysis of enriched pRBC, and the membrane containing fraction was divided into four aliquots. The first aliquot was extracted with 6 M urea, the second with 100 µM sodium carbonate at pH 11.5, the third with 1% TX-100, and the fourth with 2% SDS and 1% TX-100. Urea and sodium carbonate are commonly used to extract peripheral membrane proteins involved in protein-lipid or protein-protein interactions, whereas detergents such as TX-100 extract integral membrane proteins.

**Figure 3:**
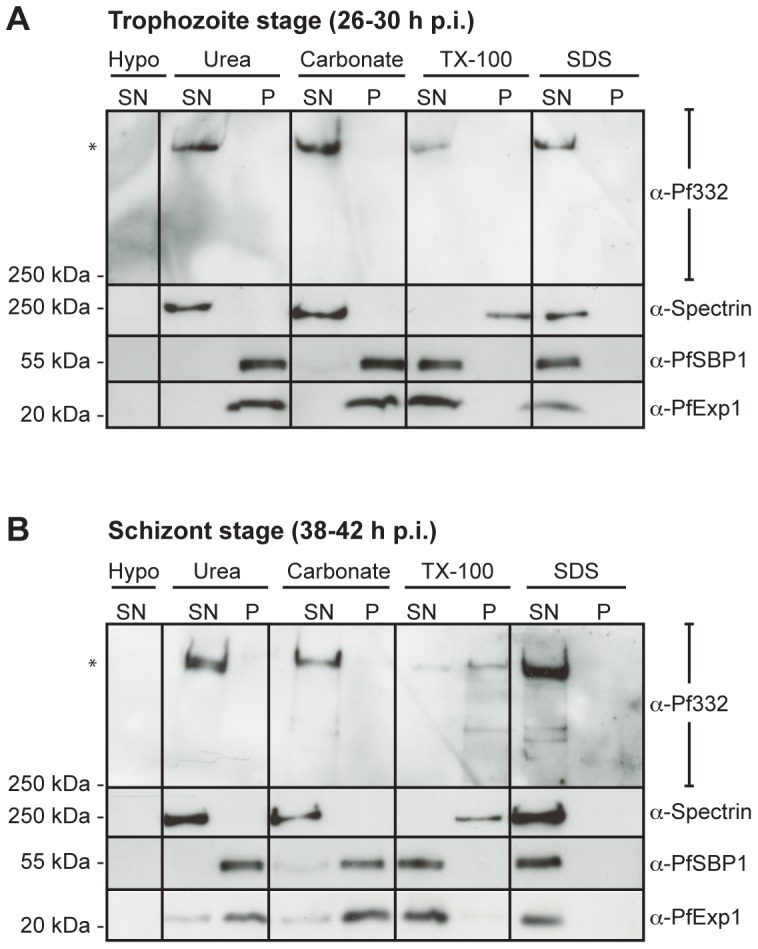
Pf332 can be extracted by both urea and sodium carbonate at its final localization in Maurer's clefts. Parasitized RBC at (**A**) 26–30 h p.i. and (**B**) and 38–42 h p.i. were lysed hypotonically. The supernatant (containing water-soluble proteins; hypo/SN) was analyzed as the soluble fraction, whereas the pellet (containing membrane proteins) was divided into four aliquots that were extracted with urea, alkaline sodium carbonate, TX-100, or SDS. Extracted material was separated by centrifugation into a supernatant/soluble fraction (SN) and a pellet/insoluble fraction (P). Extracts from 3×10^6^ pRBC were examined per lane by Western blot and membranes were probed with monoclonal anti-Pf332, monoclonal anti-spectrin, polyclonal anti-SBP1, and polyclonal anti-Exp1 antibodies. The experiment was repeated on three separate occasions, and blots shown are from the same individual experiment. Full-length Pf332 is marked with an asterisk. (SDS: sodium dodecyl sulfate).

A Western blot analysis of the different fractions revealed that hypotonic lysis of pRBC did not release any water-soluble Pf332 molecules (Figure 3A and B). This is consistent with Pf332 being a membrane associated protein and serves as a biochemical confirmation of the IFA results (Figure 1C). Moreover, Pf332 was retained in the TX-100 soluble fraction during trophozoite-stage (Figure 3A), but could mainly be found in the TX-100 insoluble fraction at schizont-stage (Figure 3B), which is in accordance with our time-course solubility assay (Figure 1B). Surprisingly, Pf332 could be extracted by both urea and alkaline sodium carbonate in trophozoite and schizont-stage pRBC (Figure 3A and B).

To validate our extraction assay, we probed membranes also with antibodies towards spectrin, SBP1, and the parasite PVM antigen Exported protein 1 (Exp1). Spectrin was solubilized by both urea and sodium carbonate, whilst it resisted extraction with TX-100, consistent with spectrin being a cytoskeleton protein that participates in protein-protein interactions (Figure 3A and B). In comparison, SBP1 displayed a typical solubility profile of an integral membrane protein [25] as it resisted hypotonic lysis and extraction by both urea and sodium carbonate and was only solubilized by TX-100 (Figure 3A and B). Similarly, Exp1 resisted extraction by hypotonic lysis, sodium carbonate, and urea, whereas it was completely solubilized by TX-100 (Figure 3A and B), supporting its integrated location in the lipid bilayer of the PVM [25], [41]. SDS was used as a positive control throughout the assay and all proteins investigated were completely extractable by SDS-treatment.

### The entire Pf332 molecule is located on the cytoplasmic side of Maurer's clefts

**Figure 4:**
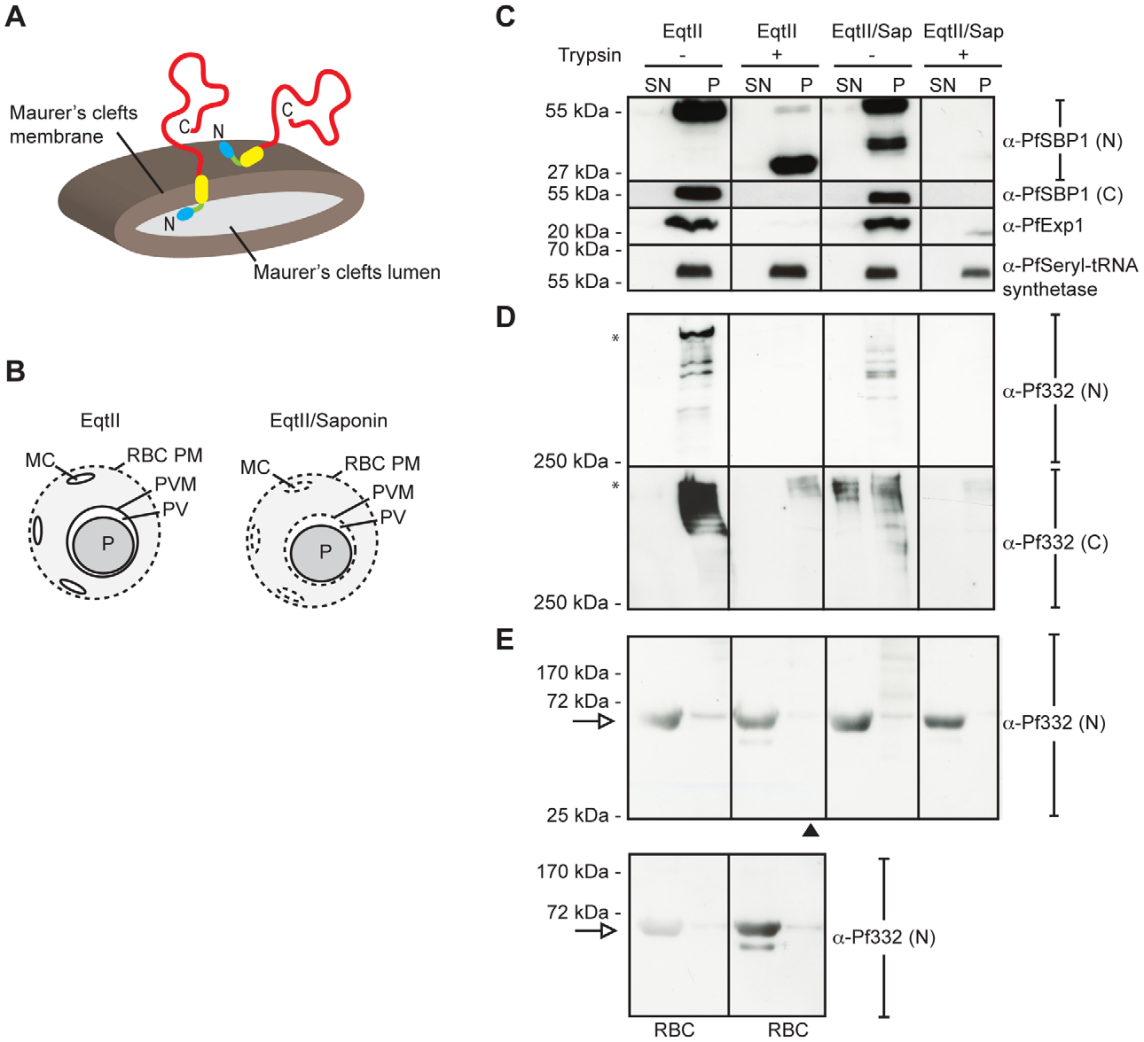
The entire Pf332 molecule associates with the cytosolic face of Maurer's clefts. (**A**) Model depicting possible membrane orientations of endogenous Pf332 in MC (left: membrane spanning protein of the clefts, right: peripheral membrane protein of the clefts). Blue: N-terminal DBL domain, yellow: predicted TM, and red: C-terminal glutamic acid-rich repeats. (**B**) Schematic mechanism of action of Equinatoxin II (EqtII) and EqtII/saponin. (**C**) *P. falciparum* pRBC at 36–40 h p.i. were differentially permeabilized with EqtII or EqtII/Sap and treated with (+) or without (−) trypsin. Supernatant (SN) and pellet (P) fractions were prepared by centrifugation and examined by Western blot. As a mean of controlling the permeabilization procedure, polyclonal antibodies against PfSBP1 (N- and C-terminus), PfExp1 (C-terminus), and PfSeryl-tRNA synthetase were used. The N-terminus of SBP1 is facing the lumen of MC, whereas the C-terminus is facing the RBC cytosol. Also the C-terminus of Exp1 is facing the RBC cytosol. Seryl-tRNA synthetase is a marker for the parasite. (**D**) The same experiment as above but probing with antibodies targeting the N-terminus (DBL domain) and the C-terminus (EB200) of Pf332. (**E**) The same experiment as above but detecting smaller sized Pf332 fragments. The upper panel represents pRBC, whereas the lower panel represents unparasitized RBC. The latter was added as a control to visualize background reactivity of the anti-Pf332 antibody, and a white arrow indicates an unspecific RBC band detected by the antibody. A black arrowhead indicates the fraction where a band representing DBL+TM would be visible if protected from trypsin digestion due to its localization within the lumen of MC. The experiment was repeated on three separate occasions, and blots shown are from the same individual experiment. In all blots, each lane represents protein extract from 2.5×10^6^ pRBC or RBC. Full-length Pf332 is marked with an asterisk. (RBC PM: red blood cell plasma membrane, MC: Maurer's clefts, PV: parasitophorous vacuole, PVM: parasitophorous vacuole membrane, P: parasite, TM: transmembrane domain).

The extractability of Pf332 in both urea and high pH sodium carbonate suggests that Pf332 is a peripheral membrane protein of Maurer's clefts and that the antigen anchors to the membrane of the clefts via either protein-protein or protein-lipid interactions. In order to verify a cytosolic and peripheral membrane localization of Pf332, we employed a biochemical approach based on the accessibility of the antigen to trypsin digestion after selectively permeabilizing membranes of enriched pRBC using the detergents EqtII and saponin [25]. EqtII selectively forms pores in the RBC plasma membrane by interacting with sphingomyelin, but leaves the PVM, Maurer's clefts membrane, and parasite plasma membrane intact [38], [39]. Saponin on the other hand disrupts the RBC plasma membrane, PVM, and Maurer's cleft membrane by interacting with cholesterol, but leaves the parasite plasma membrane intact [38]. Possible models for membrane topology of Pf332 are depicted in Figure 4A, and a schematic mechanism of action of EqtII and EqtII/saponin are shown in Figure 4B.

Parasitized RBC at 36–40 h p.i. were selectively permeabilized with EqtII alone or in combination with saponin, and treated with (+) or without (−) trypsin. To validate the procedure, we initially investigated parasite-derived antigens with a known membrane topology and subcellular localization. A Western blot analysis using antibodies targeting the N- and C-terminal domains of SBP1 revealed that the protein was exclusively associated with the pellet fractions in untreated EqtII- and EqtII/saponin-permeabilized cells. Following trypsin treatment of EqtII-permeabilized pRBC, the C-terminus was digested, whereas the N-terminus was protected resulting in a truncated product (Figure 4C). In comparison, both the N- and C-terminal domains were digested by trypsin in EqtII/saponin-permeabilized cells. These data are consistent with SBP1 being an integral membrane protein, with the N-terminus facing the lumen of Maurer's clefts and the C-terminus facing the host cell cytosol [19], [25]. Similarly, an antibody targeting the C-terminus of Exp1 detected the protein in the pellet fractions of untreated EqtII- and EqtII/saponin-permeabilized pRBC, whereas the protein was digested in trypsin-treated EqtII- and EqtII/saponin-permeabilized cells (Figure 4C), consistent with previous reports demonstrating that Exp1 resides in the PVM with the C-terminus exposed to the RBC cytosol [25], [41]. Seryl-tRNA synthetase was used as an intracellular marker of the parasite, and it was consistently found in the pellet fractions where it was inaccessible to trypsin digestion (Figure 4C), verifying that the parasite plasma membrane was left intact during the procedure.

To investigate the membrane orientation of the endogenous Pf332 molecule, we used antibodies targeting the N-terminal DBL domain and the C-terminal Glu-rich EB200 repeats (Figure 1A). Since the N-terminal domain is considerably smaller than the C-terminal region, it was not possible to detect both full-length Pf332 and potentially truncated protein on the same membrane.

We first set out to determine the subcellular localization of the massive C-terminus of Pf332. A Western blot analysis using antibodies towards both the N- and C-terminal domains of Pf332 showed retention of full-length protein within the pellet fractions following permeabilization of pRBC with EqtII or EqtII/saponin, (Figure 4D). After treatment of EqtII-permeabilized cells with trypsin, full-length Pf332 was no longer visible, implying that the massive Glu-rich C-terminus was accessible to trypsin digestion due to its localization in the RBC cytosol. Trypsin treatment of EqtII/saponin-permeabilized cells similarly resulted in digestion of Pf332. Noteworthy, the Pf332-EB200 antibody identified a small amount of soluble protein in the supernatant of pRBC permeabilized with EqtII/saponin, suggesting that a population of Pf332 was either water-soluble within the PV, or present in the host cell cytosol where it was solubilized by saponin (Figure 4D).

We next set out to determine the subcellular localization of the N-terminal DBL domain of Pf332. If the DBL domain was located in the lumen of Maurer's clefts, it would similarly to the N-terminus of SBP1 be protected from trypsin digestion in EqtII-permeabilized pRBC, whereas the C-terminal repeat region that is facing the RBC cytosol would be accessible. Accordingly, the anti-Pf332-DBL antibody would not be able to detect full-length protein in those cells, but it would be able to detect a band migrating at approximately 70 kDa (representing truncated Pf332), whereas this band would be missing in trypsin-treated EqtII/saponin-permeabilized cells. However, no truncated Pf332 protein migrating below 200 kDa was detected in EqtII-permeabilized pRBC upon trypsin treatment, and the Pf332 signal was lost completely (Figure 4E). Thus, the DBL domain was accessible to trypsin digestion, which can only occur if also the DBL domain was located in the host cell cytosol. It should be noted that a trypsin resistant band migrating at approximately 60 kDa was visible in the soluble fractions and a weak band of the same molecular weight was visible in the insoluble fractions; however, these polypeptides were not parasite-specific as they were detected also in extracts from unparasitized RBC (Figure 4E). Some additional bands of unknown origin were also present in the EqtII/saponin-treated pellet fraction; however, since trypsin was not added in this case, these cannot represent truncated Pf332 (Figure 4E). Taken together, our data verify that Pf332 does not span the membrane of Maurer's clefts, and instead argue that the antigen attaches peripherally to the cytosolic face of the clefts.

### Pf332 is synthesized and trafficked as a sodium carbonate extractable protein

**Figure 5:**
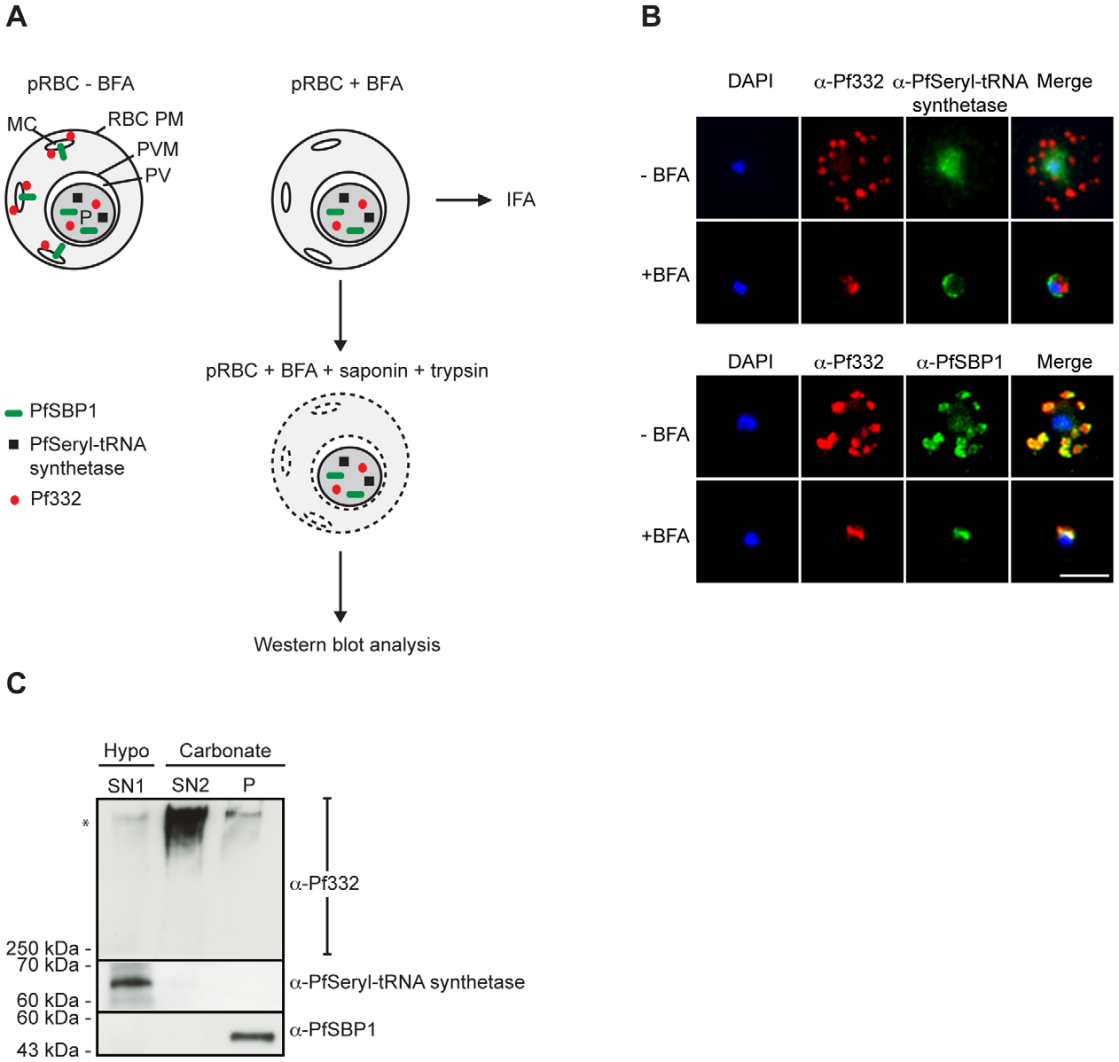
Pf332 is synthesized and trafficked within the parasite as a sodium carbonate extractable protein. (**A**) Schematic representation of the experiment. Parasitized RBC at 6–10 h p.i. were mock-treated (BFA−) or BFA treated (BFA+) for 20 h (until reaching approximately 28–32 h p.i.). To verify that BFA inhibited protein export, BFA+ and BFA− pRBC at 28–32 h p.i were collected for IFA, as described in 5B. In order to analyze in what physiological state Pf332 was synthesized and trafficked within the parasite, BFA+ pRBC at 28–32 h p.i. were lysed with saponin and trypsin-treated to remove any exported protein. Intact parasites were subsequently collected and proteins extracted, as described in 5C. (**B**) Air-dried monolayers of BFA+ and BFA− pRBC were probed with monoclonal anti-Pf332-DBL (red) and polyclonal anti-PfSeryl-tRNA synthetase antibodies (green), or polyclonal anti-Pf332-E200 (red) and polyclonal anti-PfSBP1 antibodies (green). The parasite was counterstained with DAPI (blue). Scale bar indicates 5 µm. (**C**) Intact parasites were lysed in a hypotonic buffer and the resulting protein extract was separated by centrifugation into a soluble supernatant and an insoluble pellet. The supernatant was analyzed as water-soluble proteins (SN1). The membrane fraction was extracted with alkaline sodium carbonate and the resulting protein extract was separated by centrifugation into a soluble supernatant and an insoluble pellet. The supernatant was analyzed as sodium carbonate released peripheral proteins (SN2), and the pellet was analyzed as insoluble integral membrane proteins (P). Extracts from 3×10^6^ pRBC were examined per lane by Western blotting using monoclonal anti-Pf332-DBL, polyclonal anti-PfSBP1, and polyclonal anti-PfSeryl-tRNA synthetase antibodies. Full-length Pf332 is marked with an asterisk. (BFA: Brefeldin-A, IFA: immunofluorescence microscopy assay).

We next wanted to determine whether Pf332 was trafficked within the parasite in a water-soluble state or as part of a multimeric protein complex. To determine this, we made use of the fact that export of Pf332 is sensitive to treatment with Brefeldin-A (BFA) [32], a fungal metabolite that inhibits export of proteins from the parasite by causing the Golgi complex to disassemble and redistribute to the ER [42]. Synchronous early ring-stage pRBC were treated with (+) or without (−) BFA for 20 h (experimental outline is depicted in Figure 5A), and inhibition of protein export was verified by IFA. As controls, we included antibodies towards the BFA sensitive SBP1 [19], and the non-exported Seryl-tRNA synthetase. In the absence of BFA, both the anti-Pf332 and anti-SBP1 antibodies stained Maurer's clefts, whereas the Seryl-tRNA synthetase antibodies only stained the parasite (Figure 5B). In contrast, in the presence of BFA, export of Pf332 and SBP1 was completely blocked, as demonstrated by their retention together with Seryl-tRNA synthetase within the parasite (Figure 5B).

After having confirmed the inhibitory effect of BFA on protein export, we next set out to investigate the physiological state in which Pf332 is trafficked. Intracellular parasites from BFA+ pRBC were released by treatment with saponin and any proteins that had been exported beyond the confines of the parasite prior BFA treatment were removed by trypsin digestion. Water-soluble and membrane fractions were prepared, and the latter was extracted with sodium carbonate. In the presence of BFA, both Pf332 and SBP1 were inaccessible to trypsin digestion due to their blocked export, as demonstrated by their presence in the Western blot analysis (Figure 5C). Similarly to what was observed with exported Pf332 (Figure 3), intraparasitic protein was solubilized by treatment with sodium carbonate (SN2), indicating that Pf332 was associating with internal membranes within the parasite and/or present in multimeric protein complexes during trafficking. In comparison, Seryl-tRNA synthetase was recovered in the water-soluble fraction (SN1) and SBP1 was retained in the insoluble pellet (P), in accordance with the former being a water-soluble protein and the latter being an integral membrane protein.

## Discussion

Following invasion of human RBC, *P. falciparum* dramatically modifies its host cell to suit its own needs. These modifications include the establishment of a parasite-derived trafficking machinery involving Maurer's clefts, and alterations of the host cell deformability. We here report that Pf332, the largest known exported asexual *P. falciparum* antigen, is synthesized as a peripheral membrane protein, which after export into the host cell cytosol associates with the cytoplasmic side of Maurer's clefts in a peripheral manner throughout the mature stages of parasite development. We also demonstrate that Pf332 becomes increasingly insoluble in TX-100 as the parasite matures, but that the antigen does not become exposed at the RBC surface.

Targeted gene deletions of *Pf332* have revealed that the antigen modulates host cell rigidity [33], [43]. Interestingly, although most deformability altering parasite antigens increase host cell rigidity, Pf332 appears to do the opposite [33], [43]. The underlying mechanism for this observation is unknown, but it is likely a result of interactions between Pf332 and the host cell cytoskeleton [44]. Parasite proteins that associate with the RBC cytoskeleton become insoluble in the non-ionic detergent TX-100, and this property is often used as a biochemical definition of a cytoskeleton interaction. Although a recombinant fragment of Pf332 has been shown to bind actin [44], previous biochemical analyses have brought about conflicting results concerning the solubility of endogenous Pf332 within the host cell, and the protein has been reported to be both membrane associated/TX-100 soluble [28] and cytoskeleton associated/TX-100 insoluble [33]. From both our time-course assay and extraction experiment, it was evident that the proposed interaction of Pf332 with the cytoskeleton (as determined by the insolubility of the protein in TX-100) gradually increased as the parasite matured, and that the entire Pf332 population was associating with the cytoskeleton at schizont-stage (Figure 1B). Accordingly, our data support both previous reports and demonstrate a parasite maturation-dependent interaction between Pf332 and the cytoskeleton.

Maurer's clefts are mobile in ring-stage pRBC, but become immobile and connected to the RBC actin cytoskeleton as the parasite matures [45], [46]. Moreover, they have been reported to disassemble a few hours prior merozoite egress [46]. Since Pf332 has been implicated in decreasing host cell rigidity, it is tempting to speculate that the proposed interaction between Pf332 and the RBC cytoskeleton (primarily at completion of the intraerythrocytic developmental cycle) destabilizes the RBC plasma membrane and weakens the attachment of Maurer's clefts to the cytoskeleton. It is possible that Pf332 competes with other parasite-derived proteins for specific cytoskeleton binding sites, or that the massive size and negative charge of the antigen prevent other proteins from interacting or cause them to dissociate. The resulting destabilization of the cytoskeleton may lead to an overall reduction in host cell rigidity and prepare the cell for the upcoming merozoite egress. It is worth noting that parasites with a disrupted *Pf332* gene display no striking change in multiplication rate implying that parasite egress is not permanently impaired in these parasites; however, there is most likely redundancy in protein function and other antigens could be involved in rigidity modulations in the absence of Pf332 [33].

Pf332 was early on demonstrated to be associated with Maurer's clefts [28], [32]; however, this interaction was suggested to be transient, as IFA results indicated that the antigen in very mature parasite stages relocated to the RBC plasma membrane and became exposed externally [28], [29], [30], [32]. Microscopy based localization studies of schizont-stage pRBC are problematic due to the increased permeability of the RBC plasma membrane, which can allow for antibodies to gain access to parasite proteins on the cytoplasmic face of the membrane. This can result in a staining resembling surface reactivity, leading to the conclusion of surface exposure of a molecule that is actually located in close proximity to the inner leaflet of the RBC plasma membrane. A cytoplasmic location of Pf332 is supported by the observed lack of variation in the N-terminal DBL domain, which indicates that the protein is not under any selective pressure [30]. On the basis of our IFA results (Figure 1C), it is clear that Pf332 persists in close proximity to Maurer's clefts throughout schizogony (at least until 42 h p.i.) and we therefore find it unlikely that Pf332 would translocate away from the clefts and become exposed at the surface. Furthermore, we could only detect Pf332 in schizont-stage pRBC by flow cytometry after selectively permeabilizing the RBC plasma membrane with a pore-forming cytolysin (Figure 2A). Similarly, although trypsin surface shaving of intact schizont-stage pRBC resulted in truncation of PfEMP1, Pf332 could not be cleaved in the same assay (Figure 2B). These results are consistent with previous reports [33], [43] and establish Pf332 as an intracellular molecule that is resident to Maurer's clefts.

A puzzling characteristic of the Pf332 antigen is the recently identified N-terminal DBL domain, homologous to the DBL domains of the Erythrocyte binding-like (EBL) family of invasion proteins [28]. Due to the predicted transmembrane region and the cysteine-rich nature of the DBL domain, we initially hypothesized that Pf332 is an integral membrane protein of Maurer's clefts where the DBL domain is facing the lumen of the cleft and the C-terminal repeat region is facing the host cell cytosol. A protein can typically be biochemically defined as an integral membrane protein if it can resist extraction by urea and sodium carbonate and only be extracted from the membrane after solubilization with a detergent (e.g. TX-100). In comparison, urea and sodium carbonate successfully dissolve protein complexes and extract proteins that are peripherally anchored into membranes through protein-protein or protein-lipid interactions without dissociating the lipid bilayer [47], [48]. The observation that Pf332 in Maurer's clefts can be extracted by both urea and alkaline sodium carbonate does not support Pf332 as an integral membrane protein, but instead implies that the protein is peripherally attached to the clefts (Figure 3). PfEMP1 is an unusual membrane spanning protein since it can be extracted by urea even after assuming a transmembrane topology with the N-terminus exposed at the RBC surface and the C-terminus facing the RBC cytosol [37]. PfEMP1 is therefore considered to span the RBC plasma membrane as part of a multimeric protein complex where the transmembrane domain is not directly interacting with the lipid bilayer [37]. For that reason, it was important for us to verify a cytosolic localization and peripheral attachment of Pf332 to Maurer's clefts, and rule out the possibility that the antigen spans the membrane of Maurer's clefts as part of a multimeric protein complex. Since trypsin treatment of EqtII-permeabilized cells did not result in truncation of Pf332, but rather a complete loss in signal, we can conclude that the entire antigen is accessible to trypsin digestion, which can only occur if the entire antigen resides in the host cell cytosol. We therefore suggest that Pf332 is a peripheral membrane protein of Maurer's clefts and that the predicted transmembrane domain does not serve as a membrane spanning region, but rather a hydrophobic stretch. It should be noted that although these assays strongly suggest that Pf332 is a peripheral membrane protein of Maurer's clefts, they do not specify whether the binding partner of Pf332 in Maurer's clefts is of protein or lipid origin.

Charge differences between the N- and C-termini are important in determining membrane topology of integral membrane proteins. Integral Maurer's clefts-resident proteins MAHRP1, SBP1, and REX2 all have positively charged basic C-terminal domains that are oriented towards the RBC cytosol (predicted pI: 6.2, 9.9, and 9.2, respectively), whereas their more negatively charged acidic N-terminal domains are facing the lumen of Maurer's clefts (predicted pI: 3.9, 4.7, and 5.0, respectively). In contrast, REX1, a peripheral membrane protein of Maurer's clefts [49], contains a highly negatively charged C-terminal region with an overall pI of 4.9 [49]. Interestingly, Pf332 displays more similarities with this peripheral membrane protein than with the integral membrane proteins, since Pf332 contains an extremely large acidic C-terminal domain with a predicted pI of 3.8. Interestingly, a genetic knockout of the genes encoding REX1 and Pf332 both resulted in distortion of Maurer's clefts morphology, as illustrated by a stacked and aggregated appearance of the clefts [33], [49]. This argues for that both these peripheral membrane proteins play a structural role in the maintenance of Maurer's clefts' morphology, presumably by separating the clefts into individual lamellae with their highly negatively charged C-terminal domains.

Genetically truncated Pf332 molecules containing only the first 90 kDa (including the N-terminal DBL domain and the hydrophobic stretch) have been shown to be correctly trafficked to Maurer's clefts, illustrating that both the trafficking information and membrane attachment sites of Pf332 are enclosed within this region [43]. From our solubility time-course, it was evident that Pf332 became processed and that the smaller sized Pf332 products contained both the DBL domain and the hydrophobic stretch (Figure 1B). In that particular assay, we did not separate water-soluble and TX-100 soluble proteins, and we therefore cannot say whether these processed Pf332 fragments were membrane associated or not. However, at the same time-points, Pf332 was found in close association with the membrane of Maurer's clefts by IFA (Figure 1C). Moreover, in subsequent experiments we were unable to detect any water-soluble Pf332 protein after both hypotonic lysis and EqtII permeabilization of pRBC (Figure 3 and 4D, respectively). Consequently, the processed Pf332 polypeptides appear to be membrane associated, which is in accordance with this region containing the membrane attachment site/s. Intriguingly, a GFP-chimera of Pf332 containing only the DBL domain and the hydrophobic stretch has been shown to be correctly targeted to Maurer's clefts (Osamu Kaneko et al. 2012, personal communication). Thus, we postulate that the hydrophobic stretch of Pf332 serves as a recessed signal peptide that allows the protein to enter the secretory pathway (as has previously been shown for other PEXEL negative Maurer's clefts residents [25], [50], [51]), and that the N-terminal DBL domain contains additional trafficking information that directs the antigen onwards into the RBC cytosol. However, additional studies are necessary in order to in detail characterize which regions and sequences are required for Pf332 trafficking (e.g. RSLAD). More studies are also required to determine whether the DBL domain of Pf332 can form the DBL-characteristic cysteine-bridges [43], [52], if present in a reducing cytosolic environment.

EqtII-permeabilized pRBC did not release any water-soluble Pf332 protein in the supernatant fraction; however, a small population of Pf332 was found to be soluble in EqtII/saponin-permeabilized cells (Figure 4D). This partial release upon saponin treatment indicates that either a delayed population of trafficked Pf332 was present in the PV in a water-soluble form, or that saponin was able to disrupt a small population of Pf332 that was interacting weakly with the membrane of Maurer's clefts and/or the host cell cytoskeleton. Considering that were unable to detect any water-soluble Pf332 in our extraction assay (Figure 3), we prefer the latter explanation. Indeed, it has been reported that hemolysis with saponin destabilizes the cytoskeleton and plasma membrane in RBC and leads to an increased dissociation of peripheral membrane proteins [53]. Interestingly, Külzer et al. recently demonstrated that saponin treatment of pRBC solubilized a parasite-encoded heat-shock protein 40 (Hsp40) that was tightly associated with J-dots, parasite-derived membranous structures found in the host cell cytosol [54]. The authors postulated that saponin disrupted the cholesterol organization in the J-dot membrane, resulting in release of Hsp40 into the soluble supernatant. It is possible that Pf332 interacts directly or indirectly with a membrane bound protein or lipid that is to some extent enriched in cholesterol rich sub-domains of Maurer's clefts. However, since only a small proportion of Pf332 was solubilized upon saponin treatment, it is unlikely that this is a general feature of Pf332. Of note, we could not detect any solubilized Pf332 upon saponin treatment when using the anti-Pf332-DBL antibody, however; it must be taken into consideration that the anti-Pf332-EB200 antibody (towards the repeats of the antigen) is a more potent antibody as the repeats are present over a 630 kDa region of Pf332, whilst the DBL domain is present only as a single copy domain in the 70 kDa N-terminus of the protein (Figure 1A).

Trafficking and export of parasite antigens into the host cytosol is an important part of parasite development and virulence, and a number of antigens, including Pf332, are trafficked along the classical secretory pathway as evidenced by their sensitivity to treatment with BFA [32], [55]. A GFP fusion of REX1 has been shown to be trafficked in a soluble state [51], but peripheral proteins may also be trafficked as part of a multimeric protein complex, as is the case for cytoskeleton-binding proteins MESA [56], PfEMP3 [57], and KAHRP [55]. In comparison, transmembrane proteins are usually co-translationally inserted into the lipid bilayer of the ER and trafficked as membrane embedded proteins. Interestingly, PfEMP1 is synthesized and trafficked as a sodium carbonate soluble protein, which only after reaching Maurer's clefts or the RBC plasma membrane assumes a transmembrane topology [37]. Accordingly, it has been suggested that PfEMP1 is trafficked within the parasite and the host cell cytosol as part of a multimeric protein complex, possibly involving chaperones [37], [58]. In the presence of BFA, we found Pf332 to be sodium carbonate extractable, indicating that the protein was peripherally associated with internal membranes of the parasite and/or present in multimeric protein complexes during the intraparasitic secretory pathway. This is in contrast to fellow peripheral membrane protein and Maurer's clefts resident REX1; however, given the extremely large size of Pf332, it seems reasonable to assume that correct trafficking of this massive antigen requires additional assistance by for example chaperones. Intriguingly, results obtained from a protein interaction network identified by a yeast-two hybrid screen in *P. falciparum* have suggested that Pf332 may interact with two predicted co-chaperones (PF14_0700; a hypothetical protein with a J domain, and PFB0595w; a Hsp40) [59]. An examination of the interactions exerted by PF14_0700 further indicated that it interacts with a number of membrane/exported proteins [59]. Since these two co-chaperones do not contain PEXEL motifs [60], it is possible that they assist in intraparasitic trafficking of proteins destined for export beyond the parasite, although this remains to be experimentally demonstrated.

**Figure 6:**
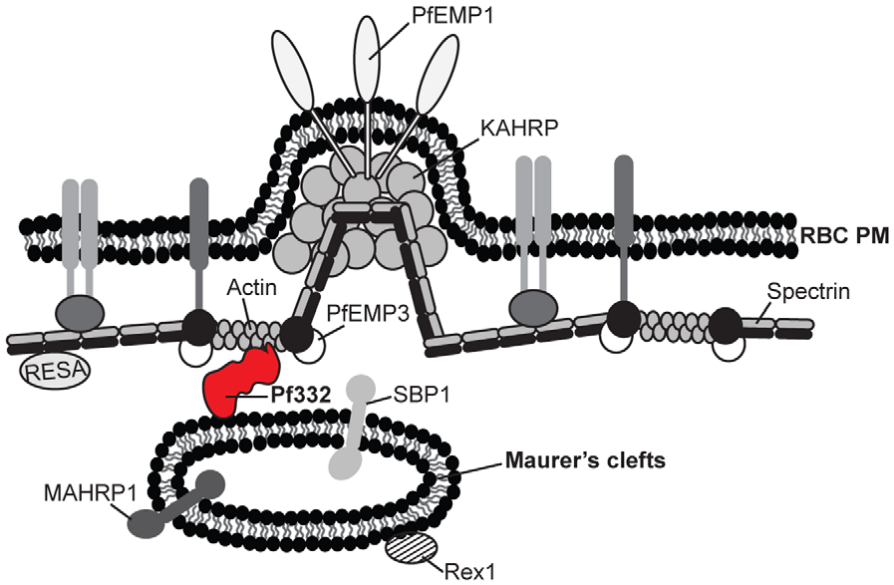
Proposed model for the subcellular localization of the Pf332 antigen. We propose that Pf332 is a resident peripheral membrane protein of Maurer's clefts, which associates with the cytoplasmic side of the clefts. At completion of the intraerythrocytic developmental cycle, Pf332 interacts with the host cell cytoskeleton. For simplicity, only antigens of relevance for this study are depicted. Pf332 is displayed in red. (RBC PM: red blood cell plasma membrane).

In conclusion, we here describe the first biochemical characterization of the endogenous Pf332 molecule from its synthesis in the ER towards its final destination in Maurer's clefts. Pf332 is not trafficked in a water-soluble state, but instead appears to be trafficked as part of a multimeric protein complex. We show that Pf332 is soluble in the non-ionic detergent TX-100 at trophozoite-stage, but becomes increasingly insoluble as the parasite matures, presumably as a result of interactions with Pf332 and the host cell cytoskeleton. Due to the nature of the N-terminal cysteine-rich DBL domain of Pf332, it has been hypothesized that the domain is either exposed at the RBC surface or located in the lumen of Maurer's clefts. We here suggest that neither of these two models are likely, and instead propose a model where the entire Pf332 antigen is located in the RBC cytosol attached to Maurer's clefts via protein-protein or protein-lipid interactions throughout trophozoite maturation and schizogony (Figure 6).

These data shed light on host cell-modifications exerted by an exported parasite antigen. Gaining more insight into the process of host-cell modifications might lead to novel intervention strategies for controlling malaria.

## Experimental Procedures

### Parasite cultures

*P. falciparum* clones HB3 and FCR3S1.2 were maintained in continuous culture according to standard procedures [61]. Briefly, parasites were cultivated at a 5% hematocrit in malaria culture medium supplemented with 10% Swedish non-immune A^+^ serum. Parasites were synchronized with sorbitol treatment.

### Triton X-100 solubility assay

HB3 pRBC at 26–30 h p.i. were enriched by magnetic cell sorting (MACS; Miltenyi Biotec, Bergisch Gladbach, Germany), which yielded a purity of approximately 90%. Parasitized cells were harvested at three time-points: 26–30 h (directly after MACS enrichment), 32–36 h, and 38–42 h p.i. (both harvested after the remaining, enriched pRBC were returned into culture to reach maturity) and each time-point contained approximately 1×10^8^ pRBC. At each time-point, pRBC were collected for immunofluorescence microscopy assays (IFA; see below). For the Western blot analysis, pRBC were divided into two fractions: one that was lysed directly in sodium dodecyl sulfate (SDS) loading buffer (representing total pRBC lysate), and one that was resuspended in 150 µl 1% Triton X-100 (TX-100) in phosphate-buffered saline (PBS) containing a complete protease inhibitor cocktail (chymotrypsin, thermolysin, papain, pronase, pancreatic extract, and trypsin; Roche, Basel, Switzerland) and frozen overnight at −80°C. After thawing, the parasite extract was incubated for 1 h at room temperature, after which TX-100 soluble proteins were separated from TX-100 insoluble proteins by centrifugation at 16 000× g for 30 min. The supernatant fractions (containing soluble proteins) were incubated at room temperature for 5 min followed by a 1 min centrifugation at 16 000× g to remove any insoluble remnants, and the pellet fractions (containing insoluble proteins) were washed twice in extraction buffer to remove any remaining soluble proteins. The pellet was subsequently resuspended in the same volume as the soluble fraction. SDS loading buffer was added to each sample, and equal amounts of protein sample were separated by SDS-polyacrylamide gel electrophoresis (SDS-PAGE) and analyzed by Western blot.

### Indirect immunofluorescence microscopy assay for detection of Pf332

For the IFA, monolayers of HB3 pRBC were prepared as previously described [31]. Slides were blocked for 1 h with 3% bovine serum albumin (BSA) in PBS and subsequently incubated for 1 h with monoclonal mouse anti-Pf332-DBL (1∶100), polyclonal rabbit anti-Pf332-EB200 (1∶500, gift from Klavs Berzins), polyclonal rabbit anti-PfMAHRP1 (1∶200, gift from Hans-Peter Beck), polyclonal mouse anti-PfSBP1 (1∶500, gift from Catherine Braun-Breton), or polyclonal rat anti-PfSeryl-tRNA synthetase (1∶250) diluted in blocking buffer. Slides were thereafter washed three times with PBS and primary antibodies were detected by incubation for 1 h with secondary ALEXA-Fluor 594-conjugated anti-mouse or anti-rabbit IgG, and ALEXA-Fluor 488-conjugated anti-mouse, anti-rabbit or anti-rat IgG (dilution 1∶1000; Invitrogen, Carlsbad, CA, USA). To avoid photobleaching, cells were mounted with Vectashield (Vector Laboratories, Burlingame, CA, USA) containing 1.5 µg/ml 4′,6-diamidino-2-phenylindole (DAPI) to stain for parasite DNA. All incubations were performed at room temperature in a humid chamber. Cells were viewed with a 100× oil immersion objective in a UV equipped Nikon Eclipse 90i/80i microscope (Nikon Corporation, Japan) and images were captured by a Hamamatsu Orca-ER CCD Digital camera (Hamamatsu Photonics System, Japan) and MicroManager software version 1.3 (developed by Arthur Edelstein, Nico Stuurman and Nenad Amodaj, University of California, San Francisco, USA). All images were finally processed using ImageJ software version 1.43 (National Institutes of Health, Bethesda, USA).

### Flow cytometry to detect surface fluorescence

FCR3S1.2 pRBC at approximately 38–42 h p.i. were used for the flow cytometry experiments. 1.5–2×10^8^ pRBC were treated with 1 µg (1 hemolytic unit) of Equinatoxin II (EqtII; gift from Gregor Anderluh) or mock-treated in PBS. Parasitized RBC were subsequently incubated in duplicates for 30 min at room temperature with monoclonal mouse anti-Pf332-DBL, monoclonal mouse anti-PfEMP1-NTSDBL1α or non-immune mouse IgG (Jackson ImmunoResearch, West Grove, PA, USA) at a concentration of 50 µg/ml, or with monoclonal mouse anti-PfEMP1-ATS, rabbit anti-Pf332-EB200, rat anti-Pf332-DBL antibodies or rat/rabbit pre-bleed at a 1∶20 dilution. After incubation with the primary antibody, pRBC were washed in PBS with 2% fetal calf serum (FCS) followed by 30 min incubation with secondary ALEXA-488 coupled anti-rat, anti-mouse or anti-rabbit IgG (dilution 1∶100, Invitrogen). For nuclear staining, ethidium bromide was added to a final concentration of 2.5 µg/ml. The pRBC were washed twice and resuspended in PBS with 2% FCS. Cell acquisition was done using flow cytometry (FACSCalibur, BD Bioscience, http://www.bd.com) where 5000 pRBC were counted. The analysis was performed using the FlowJo software (FlowJo, Ashland, OR, USA).

### Trypsin digestion assay for detection of surface proteins

The trypsin digestion assay was performed as described elsewhere [40]. Briefly, 38–42 h p.i. pRBC were treated with 1 mg/ml trypsin (Sigma) or mock-treated in PBS for 1 h at 37°C. The reaction was stopped by adding soybean trypsin inhibitors (to a final concentration of 2 mg/ml). Cells were subsequently washed in PBS and resuspended in SDS-loading buffer and equal amounts of protein sample were separated by SDS-PAGE and analyzed by Western blot.

### Differential extraction of proteins

HB3 pRBC at 26–30 h p.i. were enriched by MACS and harvested at two time-points: 26–30 h (directly after MACS enrichment) and 38–42 h p.i. (after the remaining enriched pRBC had been returned into culture to reach maturity). 1.4×10^8^ pRBC were resuspended in 200 µl 7.5 mM Tris-HCl pH 8.0, containing a complete protease inhibitor cocktail and frozen overnight at −80°C. After thawing, the lysate was centrifuged at 100 000× g for 60 minutes in order to separate soluble aqueous proteins from insoluble proteins. The supernatant fraction (containing water-soluble proteins) was incubated at room temperature for 5 min followed by another centrifugation to remove any insoluble remnants. The pellet (containing membrane proteins) was washed twice in 7.5 mM Tris-HCl (pH 8.0) and divided into four aliquots. The first aliquot was extracted with 200 µl 6 M urea in 7.5 mM Tris-HCl (pH 8.0) for 1 h at room temperature, the second with 200 µl 100 mM sodium carbonate (pH 11.5) for 1 h on ice, the third with 200 µl 1% TX-100 in PBS for 1 h at room temperature, and the fourth with 200 µl 2% SDS and 1% TX-100 in PBS for 1 h at room temperature. Samples were subsequently centrifuged at 100 000× g for 60 min to separate soluble and insoluble proteins. The supernatant fractions (containing solubilized proteins) were incubated at room temperature for 5 min followed by 30 min centrifugation to remove any insoluble remnants, and the pellet fractions (containing insoluble proteins) were washed twice in extraction buffer to remove any remaining soluble proteins. The pellet fractions were subsequently resuspended in the same volume as the soluble fractions. SDS-loading buffer was added to each sample, and equal amounts of sample were separated by SDS-PAGE and analyzed by Western blot.

### Trypsin digestion assay for detection of membrane topology

HB3 pRBC at approximately 24–28 h p.i. were enriched by MACS, returned into culture and collected at 36–40 h p.i. In total, 1.5–2×10^8^ pRBC were incubated with 2 µg (2 hemolytic units) of EqtII in a final volume of 120 µl RPMI-1640 for 12 min at 37°C. Cells were centrifuged for 1 min at 1000× g. After washing with 1% BSA in PBS, the pellet was divided into four equal aliquots. The first and second aliquots were resuspended in 50 µl PBS and 50 µl PBS containing 1 mg/ml trypsin, respectively. The third and fourth aliquots were resuspended in 50 µl 0.09% saponin in PBS and 50 µl 0.09% saponin in PBS containing 100 µg/ml trypsin, respectively. Samples were incubated at 37°C for 20 min and reactions were stopped by adding soybean trypsin inhibitors (to a final concentration of 2 mg/ml) and a cocktail of protease inhibitors. Samples were subsequently separated by centrifugation (5 min 16 000× g) into supernatant and pellet fractions and the pellet was resuspended in the same volume as the soluble fractions. SDS-loading buffer was added to each sample, and equal amounts of sample were separated by SDS-PAGE and analyzed by Western blot.

### Brefeldin-A treatment

Synchronous HB3 pRBC (at 6–10 h p.i.) from a 20 ml culture at 10% parasitemia were cultivated for approximately 20 h with 5 µg/ml Brefeldin-A (BFA+) from a 5 mg/ml ethanol stock solution (Sigma). Control parasites were cultivated with ethanol alone to exclude any morphological alterations due the presence of ethanol (BFA−). Parasitized cells were grown until they reached 28–32 h p.i. after which a small sample was taken for IFA, as previously described. The remaining BFA+ culture was thereafter collected by centrifugation and lysed with saponin. Intact parasites were treated with 100 µg/ml trypsin for 15 min at 37°C and the protease reaction was stopped, as previously described. Parasites were subsequently lysed by hypotonic shock in 150 µl 7.5 mM Tris-HCl (pH 8.0) containing a complete protease inhibitor cocktail and frozen overnight at −80°C. After thawing, the parasite lysate was centrifuged at 100 000× g for 60 min to separate water-soluble proteins from insoluble membrane proteins. The supernatant (containing water-soluble proteins; SN1) was incubated for 5 min at room temperature and centrifuged again to remove any remaining insoluble particles. The pellet (containing membrane proteins) was washed once in 7.5 mM Tris-HCl (pH 8.0) to clear it from contaminating insoluble particles, and thereafter extracted with 150 µl 100 mM sodium carbonate, pH 11.5, for 1 h on ice, followed by centrifugation at 100 000× g for 60 min to separate soluble and insoluble proteins. Both the supernatant (SN2) and the pellet (P) were centrifuged again to remove any contaminating remnants. The pellet fraction was resuspended in the same volume as the soluble fraction. SDS-loading buffer was added to each sample, and equal amounts of sample were separated by SDS-PAGE and analyzed by Western blot.

### Western blotting

When analyzing the Pf332 protein, equal amounts of parasite extracts (2.5×10^6^–3.3×10^6^ pRBC/lane) were separated by SDS-PAGE using a 6% separation gel and transferred onto nitrocellulose membranes (Bio-Rad, Hercules, CA, USA). For all other proteins, a 10% separation gel was used. Membranes were probed with polyclonal mouse anti-PfSBP1 N- or C-terminus (1∶1000; gifts from Catherine Braun-Breton), polyclonal rabbit anti-PfExp1 C-terminus (1∶800; gift from Klaus Lingelbach and Stefan Baumeister), polyclonal rabbit anti-Pf332-EB200 (1∶1000), polyclonal rat anti-PfSeryl-tRNA synthetase (1∶100), monoclonal mouse anti-PfEMP1-ATS (1∶250), monoclonal mouse anti-spectrin (1∶4000) or monoclonal mouse anti-Pf332-DBL (1∶100). Detection by enhanced chemiluminescence (ECL plus Western blotting detection reagents, GE Healthcare) was performed after a secondary probe of the appropriate HRP-coupled antibodies (1∶5000, GE Healthcare) were added.

## Supporting Information

Figure S1 Pf332 is not exposed at the red blood cell surface. Surface expression time-course of live intact HB3 pRBC by flow cytometry. To detect Pf332, monoclonal mouse anti-Pf332-DBL and polyclonal rat anti-Pf332-DBL (N-terminus of Pf332) antibodies were used (displayed in blue). Non-immune mouse IgG and pre-immune rat sera were used as negative controls (displayed in red).
(TIF)
