# Supplementary figures and images for: Plasmodium falciparum Antigen 332 Is a Resident Peripheral Membrane Protein of Maurer's Clefts
Source: PLoS One. 2012 Nov 20;7(11):e46980. doi: 10.1371/journal.pone.0046980 (PMC3502387; doi:10.1371/journal.pone.0046980)

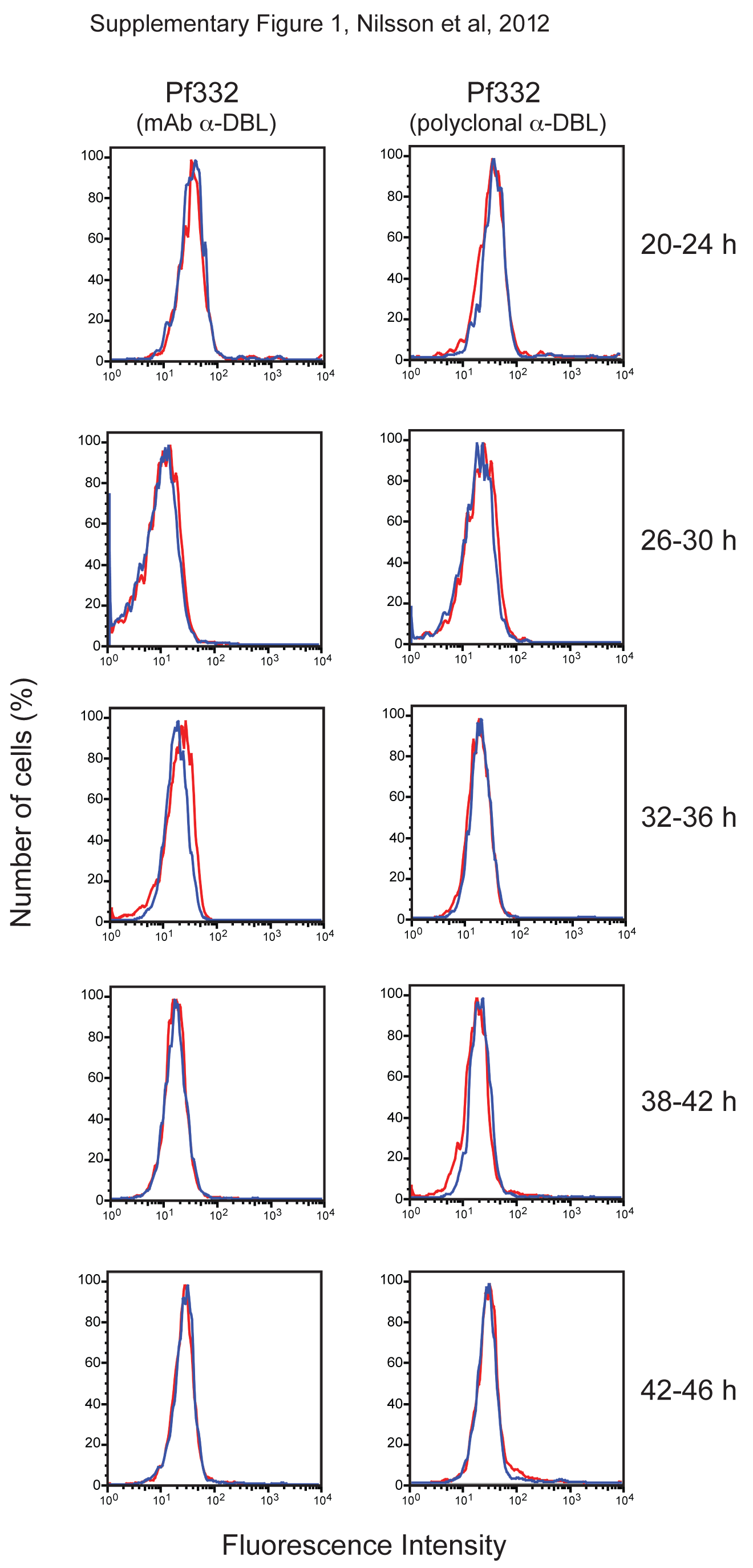

Supplement: Figure S1 — Pf332 is not exposed at the red blood cell surface. Surface expression time-course of live intact HB3 pRBC by flow cytometry. To detect Pf332, monoclonal mouse anti-Pf332-DBL and polyclonal rat anti-Pf332-DBL (N-terminus of Pf332) antibodies were used (displayed in blue). Non-immune mouse IgG and pre-immune rat sera were used as negative controls (displayed in red). (TIF) [file pone.0046980.s001.tif]
